# Interactions between antifungals and everolimus against *Cryptococcus neoformans*

**DOI:** 10.3389/fcimb.2023.1131641

**Published:** 2023-03-21

**Authors:** Pin Liang, Jiquan Song, Qin Liu

**Affiliations:** Department of Dermatology, Zhongnan Hospital of Wuhan University, Wuhan, Hubei, China

**Keywords:** *Cryptococcus neoformans*, amphotericin B, azoles, everolimus, synergistic

## Abstract

Cryptococcus is the causal agent of cryptococcosis, a disease with high mortality mainly related to HIV immunosuppression and usually manifests with pneumonia and/or meningoencephalitis. There are very few therapeutic options; thus, innovative approaches are required. Herein, We examined the interaction of everolimus (EVL) with amphotericin B (AmB) and azoles [fluconazole (FLU), posaconazole (POS), voriconazole (VOR), itraconazole (ITR)] against *Cryptococcus*. Eighteen *Cryptococcus neoforman* clinical isolates were analyzed. Following the guidelines of the Clinical and Laboratory Standards Institute (CLSI) M27-A4, we conducted a broth microdilution experiment to determine the minimum inhibitory concentrations (MICs) of azoles, EVL, and AmB for assessing antifungal susceptibility. A fractional inhibitory concentration index (FICI) of less than and equal to 0.5 indicated synergy, with a range of 0.5 to 4.0 indicated indifference and a value more than 4.0 indicated antagonism. These experiments revealed that EVL had antifungal activity against *C. neoforman*. Moreover, EVL, POS, AmB, FLU, ITR, and VOR exhibited MIC values ranging from 0.5-2 μg/mL, 0.03125-2 μg/mL, 0.25-4 μg/mL, 0.5-32μg/mL, 0.0625-4μg/mL and 0.03125-2μg/mL, respectively. The combination of EVL with AmB and azoles (POS, FLU, ITR, and VOR) exhibited synergistic antifungal effects against 16 (88.9%), 9 (50%), 11 (61.1%), 10 (55.6%) or 6 (33.3%) of analyzed *Cryptococcus* strains. In the presence of EVL, the MIC values of AmB and azoles were significantly lowered. No antagonism was observed. Subsequently, *in vivo* analyses conducted using the *G. mellonella* model further confirmed that combination EVL+ POS, EVL+ FLU, and EVL+ITR treatment were associated with significantly improved larval survival following *Cryptococcus* spp. infection. These findings provide the first published evidence suggesting that a combination of EVL and AmB or azoles exhibit a synergistic effect and may be an effective antifungal disease treatment strategy for infections caused by *Cryptococcus* spp.

## Introduction

Cryptococcosis generally occurs as an opportunistic infection in immunocompromised hosts. Patients with acquired immune deficiency syndrome (AIDS) or other immune deficiencies are especially at risk (Pappas et al., 2001; Singh and Forrest, 2009; DiNardo et al., 2015; Henao-Martínez and Beckham, 2015). *Cryptococcus*, as the causative agent of cryptococcosis, is found in various environmental sources, including contaminated milk, birds droppings, and soil. Pneumonia, meningitis, skin, soft tissue, bone, and joint infections (Cho et al., 2021) are typical cryptococcal symptoms; however, the infection may spread to other organs *via* the lymphatic system or the circulatory system.

Globally, cryptococcosis is one of the deadliest invasive mycoses due to its high morbidity and death rate (Perfect et al., 2010). About 200,000 individuals a year are killed by pathogenic species of *Cryptococcus* (Fonseca et al., 2019). Standard therapy is a typically an aggressive intravenous injection of an antifungal drug, followed by suppressive treatment taken orally for a period that varies depending on the patient’s condition (Cho et al., 2021). Amphotericin B (AmB) (typically lipid formulations) plus 5-fluorocytosine (5FC) for induction treatment for 2 weeks, followed by fluconazole as suppressive therapy, is the recommended treatment (Perfect et al., 2010; Cho et al., 2021). However, the high dosages needed for these infections, the severe toxicities of AmB have been a limiting factor in its use (Thakur and Revankar, 2011). Furthermore, in regions with higher disease load and death rates, the availability of 5-FC is limited (Maziarz and Perfect, 2016). Cryptococcosis remains a challenging management issue. Combination therapy with drug repositioning has been seen as a potential option due to the scarcity of novel antifungal medicines.

Everolimus (EVL), an analog of the naturally occurring macrolide rapamycin, is an orally bioactive inhibitor of the mammalian target of rapamycin (mTOR) serine/threonine kinase signal transduction pathway, which controls proliferation, cell growth, and survival. Its ability to directly inhibit tumor proliferation and cell growth and indirectly impede angiogenesis has garnered much interest as an anticancer drug (Hasskarl, 2018). TOR was first discovered in the yeast *Saccharomyces cerevisiae*, and subsequent research has shown that it is present in many other eukaryotic organisms, including plants, worms, flies, fungi, humans, and mammals (Crespo and Hall, 2002). Immunosuppressive medicines increase the likelihood of developing invasive fungal infections, although they also have antifungal activity. Rapamycin has shown intrinsic antifungal activity against *Candida albicans*, *Microsporum gypseum*, *Trichophyton granulosum* (Vézina et al., 1975), *Aspergillus* spp.(High and Washburn, 1997), *Fusarium fujikuroi*(Teichert et al., 2006)and *cryptococcus neoformans*(Cruz et al., 1999). Moreover, Manish Thakur et al. demonstrated a synergistic effect of caspofungin with rapamycin against *Glomeromycetes*(Thakur and Revankar, 2011). In addtion, Patrick Schwarz et al. observed that rapamycin may improve the efficacy of isavuconazole against *Aspergillus* species *in vitro* (Schwarz and Dannaoui, 2020). Despite this, EVL’s effectiveness against *Cryptococcus* has only been partially studied by itself or in conjunction with other treatments.

The rapalog effect of EVL against clinical *Cryptococcus* isolates was validated *in vitro* using AmB and azoles. To further investigate the potential treatment-related changes in larval survival, we extended these experiments to evaluate the effects of combination EVL+AmB or azoles therapy on *Galleria mellonella* larvae infected with *Cryptococcus* spp.

## Materials and methods

### Fungal strains and preparation of conidia

18 clinical *Cryptococcus neoformans* isolates were studied in total (**Table 1**). Microscopic examination, and the internal transcribed spacer (ITS) ribosomal DNA (rDNA) sequencing and D1/D2 were used to confirm *Cryptococcus* spp. identification. *Cryptococcus* conidia were collected by flooding the culture surface with phosphate-buffered saline (PBS) after being cultured at 37°C for 2 days on Potato dextrose agar (PDA) and then counted using a hemocytometer.

**Table 1:** MICs and FICIs results with combinations of Everolimus with antifungal agents against *Cryptococcus neoformans*.

Strain	MIC (µg/ml)						MICs of drug A/drug B (µg/ml), or FICI (susceptibility)				
	EVL	POS	FLU	ITR	VOR	AmB	EVL/POS	EVL/FLU	EVL/ITR	EVL/VOR	EVL/AmB
05781	1	0.03125	0.5	0.125	0.03125	4	0.0625/0.03125 (1.0625, I)	0.0625/0.5 (1.0625, I)	0.0625/0.0625 (0.5625, I)	0.0625/0.03125 (1.0625, I)	0.25/0.0625 (0.265625, S)
05338	1	0.5	0.5	0.0625	0.03125	4	0.125/0.03125 (0.1875, S)	0.0625/0.5 (1.0625, I)	0.0625/0.0625 (1.0625, I)	0.0625/0.03125 (1.0625, I)	0.25/0.125 (0.28125, S)
07190	1	0.0625	4	0.125	0.0625	2	0.0625/0.03125 (0.5625, I)	0.0625/0.5 (0.1875, S)	0.5/0.0625 (1, I)	0.0625/0.03125 (0.5625, I)	0.25/0.0125 (0.25625, S)
07394	2	0.125	2	0.0625	0.0625	4	0.0625/0.03125 (0.28125, S)	0.5/0.0625 (0.28125,S)	0.0625/0.0625 (1.03125, I)	0.0625/0.03125 (0.53125, I)	0.5/0.25 (0.3125, S)
07746	1	0.125	2	0.125	<0.03125	2	0.125/0.03125 (0.375, S)	0.125/0.5 (0.375, S)	0.5/0.0625 (1, I)	1/0.03125 (>2, I)	0.0625/0.125 (0.125, S)
07789	1	<0.03125	<0.5	0.125	0.03125	1	1/0.03125 (>2, I)	1/<0.5 (2, I)	0.0625/0.0625 (0.5625, I)	1/<0.03125 (1< <2, I)	0.125/0.5 (0.625, I)
07906	1	0.125	16	4	2	4	0.5/0.0625 (1, I)	0.5/0.5 (0.53125, I)	0.125/1 (0.375, S)	0.5/0.03125 (0.515625, I)	0.25/0.25 (0.3125, S)
05009	1	0.5	8	4	0.125	2	0.25/0.03125 (0.3125, s)	0.0625/1 (0.1875, S)	0.0625/0.0625 (0.078125, S)	0.25/0.03125 (0.5, S)	0.125/0.25 (0.25, S)
08026	1	0.25	16	0.25	0.125	4	0.25/0.03125 (0.375,S)	0.25/1 (0.3125, S)	0.0625/0.0625 (0.3125, S)	0.125/0.0625 (0.625, I)	0.125/0.25 (0.1875, S)
08061	2	0.0625	4	0.5	0.0625	2	0.125/0.03125 (0.5625,I)	0.125/0.5 (0.1875,S)	0.125/0.125 (0.3125, S)	0.25/0.03125 (0.625, I)	0.5/0.0625 (0.28125, S)
G5	1	2	16	1	0.5	4	0.125/0.5 (0.375,S)	0.125/1 (0.1875, S)	0.125/0.0625 (0.1875, S)	0.25/0.0625 (0.375, S)	0.25/0.125 (0.28125, S)
G7	1	0.25	4	2	2	4	0.0625/0.0625 (0.3125,S)	0.125/0.5 (0.25, S)	0.0625/0.5 (0.3125,S)	0.25/0.03125 (0.265625,S)	0.25/1 (0.5, S)
G8	2	0.125	8	1	0.125	1	0.125/0.0625 (0.5625,I)	1/0.5 (0.5625, I)	0.5/0.0625 (0.3125, S)	0.25/0.3125 (2.625, I)	0.5/0.0625 (0.3125, S)
G9	2	0.125	8	0.125	0.125	0.5	0.0625/0.0625 (0.53125,I)	0.5/0.5 (0.3125, S)	0.25/0.0625 (0.625,I)	0.25/0.0625 (0.625, I)	0.25/0.125 (0.375, S)
G10	1	1	16	4	0.25	4	0.0625/0.25 (0.3125,S)	0.0625/2 (0.1875, S)	0.25/0.0625 (0.265625, S)	0.5/0.03125 (0.625, I)	0.125/0.0625 (0,140625, S)
G12	2	0.5	16	1	0.5	4	0.125/0.0625 (0.1875,S)	0.25/0.5 (0.15625,S)	0.25/0.125 (0.25, S)	0.125/0.0125 (0.0875, S)	0.5/0.125 (0.28125, S)
Z2	0.5	0.25	8	0.5	0.125	0.25	0.25/0.0625 (0.75, I)	0.25/0.5 (0.5625, I)	0.25/0.125 (0.75, I)	0.125/0.03125 (0.5. S)	0.25/0.125 (1, I)
Z3	0.5	0.25	32	1	0.25	0.5	0.25/0.03125 (0.625,I)	0.25/0.5 (0.515625,I)	0.0625/0.0625 (0.1875, S)	0.125/0.0625 (0.5, S)	0.0625/0.0625 (0.25, S)

EVL, everolimus; AmB, amphotericin B; ITR, itraconazole; POS, posaconazole; FLU, fluconazole; VOR, voriconazole; MIC, minimal inhibitory concentration; FICI, fractional inhibitory concentration index; S, synergy (FICI of ≤ 0.5); I, no interaction (indifference) (0.5 < FICI ≤ 4).

### Antifungals and chemical agents

The drugs, including posaconazole (POS; purity ≥ 99%), itraconazole (ITR; purity ≥ 99%), fluconazole (FLU; purity ≥ 99%), voriconazole (VOR; purity ≥ 99%), and amphotericin B (AmB; purity ≥ 80%) were bought form from SelleckChemicals, TX, USA, in powder form. Moreover, Everoliums (EVL; purity ≥ 99%) were purchased from Shanghai Yeasen Biotechnology Co., Ltd., China. All tested agents were diluted using dimethyl sulfoxide (DMSO) as stock solutions (EVL, 6600 μg/ml; azoles and AmB, 6400 μg/ml).

### Broth microdilution assay

All 18 of the aforementioned *Cryptococcus* isolates and *Candida parapsilosis* (ATCC 22019) were used to assess the effects of EVL alone and in combination with azoles and AmB. All susceptibility testing for *Cryptococcus* spp. was performed per Clinical and Laboratory Standards Institute document M27-A4 (Clinical and Laboratory Standards Institute, 2017).The MICs were calculated as the concentrations required to limit growth by 50% (azoles) and 100% (AmB) (Clinical and Laboratory Standards Institute, 2017). The MIC values for EVL, POS, AmB, FLU, ITR and VOR were determined separately before any combination experiments were conducted. A total of 100μl of the inoculum suspension and 100μl of serial diluent of test drugs were used to inoculate a 96-well plate. Results were interpreted after incubation at 35°C for 48h for *Cryptococcus* spp. All tests were performed in duplicate.

Furthermore, the microdilution chequerboard method was used to investigate EVL’s interactions with antifungal drugs against all strains. On a 96-well plate containing 100 μl of prepared inoculum suspension, 50 μl of EVL with serial dilutions were inoculated horizontally, and 50μl of azoles or AmB with serial dilutions were inoculated vertically, as specified. Results were interpreted after incubation at 35^°^C for 48h for *Cryptococcus* spp.

FICI was determined to characterize the interaction of EVL with azoles, or AmB as follows: FICI= (Ac/Aa) +(Bc/Ba), where Ac and Bc are the MIC values for the combination of these medications and Aa and Ba are the MIC values for the drugs when taken alone. FICI of ≤ 0.5, synergy; FICI of 0.5 to ≤ 4, indifference; and FICI of > 4, antagonism (Odds, 2003). All analyses were performed in duplicate.

### *Galleria mellonella* assay

According to the methods described previously (Mylonakis et al., 2005; Amorim-Vaz et al., 2015; Liu et al., 2017), *G. mellonella* larvae were used and split into 14 different experimental

groups: untreated (noninfected larvae hat received no treatments), saline (10 µL saline injected noninfected larvae), conidial (*C. neoforman*-infected larvae), POS (200 μg/mL)-treated (treatment of *C. neoforman*-infected larvae with POS), ITR (200 μg/mL)-treated (treatment of *C. neoforman*- infected larvae with ITR), VOR (200 μg/mL)-treated (treatment of *C. neoforman*-infected larvae with VOR), FLU (200 μg/mL)-treated (*C. neoforman*-infected larvae treated with FLU), EVL (200 μg/mL)-treated (*C. neoforman*-infected larvae treated with EVL), AmB (200 μg/mL)-treated (*C. neoforman*-infected larvae treated with AmB), and POS (200 μg/mL) + EVL (200 μg/mL)-treated (treatment of *C. neoforman*-infected larvae with POS and EVL) groups, ITR (200 μg/mL) + EVL (200 μg/mL)-treated (treatment of *C. neoforman* -infected larvae with ITR and EVL) groups, VOR (200 μg/mL) + EVL (200 μg/mL)-treated (treatment of *C. neoforman-*infected larvae with VOR and EVL) groups, FLU (200 μg/mL) + EVL (200 μg/mL)-treated (treatment of *C. neoforman*-infected larvae with FLU and EVL) groups, AmB(200 μg/mL) + EVL (200 μg/mL)-treated (treatment of *C. neoforman*-infected larvae with AmB and EVL) groups. Each experimental group had 20 larvae (weighing between 0.3 and 0.4 g) included and the tests were conducted three times. All *in vivo* studies used a single *C. neoformans* isolate (G7). *C. neoformans* G7 conidia were counted using a hemocytometer at 10^6^ CFU/ml after a 2-day culture on PDA at 28°C, after which the agar was rinsed with PBS. After incubating *G. mellonella* larvae at 37°C for 2 hours, all groups except the untreated and saline control groups received an injection of 10 µL of a conidial solution and were treated with appropriate antifungal medicines (5 µl). The larvae were placed in a 37°C incubator and inspected once a day for six days to determine their survival rate.

### Statistical analysis

The statistical analysis and figure preparation were done using GraphPad Prism 5.0. Kaplan-Meier curves and log-rank (Mantel-Cox) tests were used to analyze the survival data for *G. mellonella* at a significance level of P < 0.05.

## Results

### *In vitro* antifungal activity of the individual tested agent

The MIC ranges of the individual tested agents against *Cryptococcus* isolates were 0.5-2 µg/ml for EVL, 0.03125-2µg/ml for POS, 0.25-4µg/ml for AmB, 0.5-32µg/ml for FLU, 0.0625-4 µg/ml for ITR and 0.03125-2µg/ml for VOR (**Table 1**). EVL individually showed a substantial antifungal effect against all tested strains of *Cryptococcus* spp.

### *In vitro* interactions between EVL and AmB or azoles against *C.neoformans*

Synergistic effects against 9 (50%) strains of *C. neoformans* were shown when EVL was combined with POS, and the MICs of EVL and POS against *Cryptococcus* spp. were reduced to 0.0625-1 μg/ml and 0.03125-0.5 μg/ml, respectively (**Table 1**).

The MICs of EVL and FLU against *Cryptococcus* spp. were reduced to 0.0625-1 μg/ml and 0.0625-2 μg/ml, respectively, when used in combination (**Table 1**). The synergistic effects of EVL and FLU were favorable against 11 (61.1%) strains of *Cryptococcus* spp.

The combination of EVL and ITR reduced the MICs of both drugs against *Cryptococcus*

spp. to 0.0625-0.5 μg/ml and 0.0625-1 μg/ml, respectively. When used together, EVL and VOR lowered the MICs of both against *Cryptococcus* spp. to 0.0625-1 μg/ml and 0.0125-0.3125 μg/ml, respectively. There were 10 (55.6%) and 6 (33.3%) *Cryptococcus* spp. strains that showed synergistic effects when treated with EVL/ITR and EVL/VOR, respectively (**Table 1**).

The EVL/AmB combination revealed good synergistic effects against 16 (88.9%) strains of *Cryptococcus* isolates (**Table 1**), where the MIC ranges of EVL and AmB decreased to 0.0625–0.5μg/ml and 0.0625–1μg/ml, respectively. Antagonism was never observed with AmB or azoles in combination with EVL.

### Efficacy of EVL alone and in combination with AmB or Azoles in *C.neoformans* -infected G. mellonella

Next, using *G. mellonella* larvae infected with the *C. neoformans* G7 isolate as a model system, we investigated the *in vivo* antifungal effectiveness of EVL with AmB or azoles in combination with one another or isolation. Compared to monotherapy (POS, 56.7%; FLU, 50%; ITR, 48.3%; EVL, 55%) or infected but untreated larvae (5%), survival rates were greater in the ELV+POS treatment group (66.7%), ELV+FLU treatment group (60%), and ELV+ITR treatment group (61.7%) on day 2 post-infection.

Treatment combinations of EVL and POS (58.3%), ELV and FLU (50%), and ELV and ITR (51.7%) resulted in greater larval survival on day 4 post-infection compared to monotherapy (POS: 41.7%; FLU: 45%; ITR: 36.7%; EVL,: 43.3%). The conidia-infected group with no treatment had a survival rate of 0.

**Figure 1:**
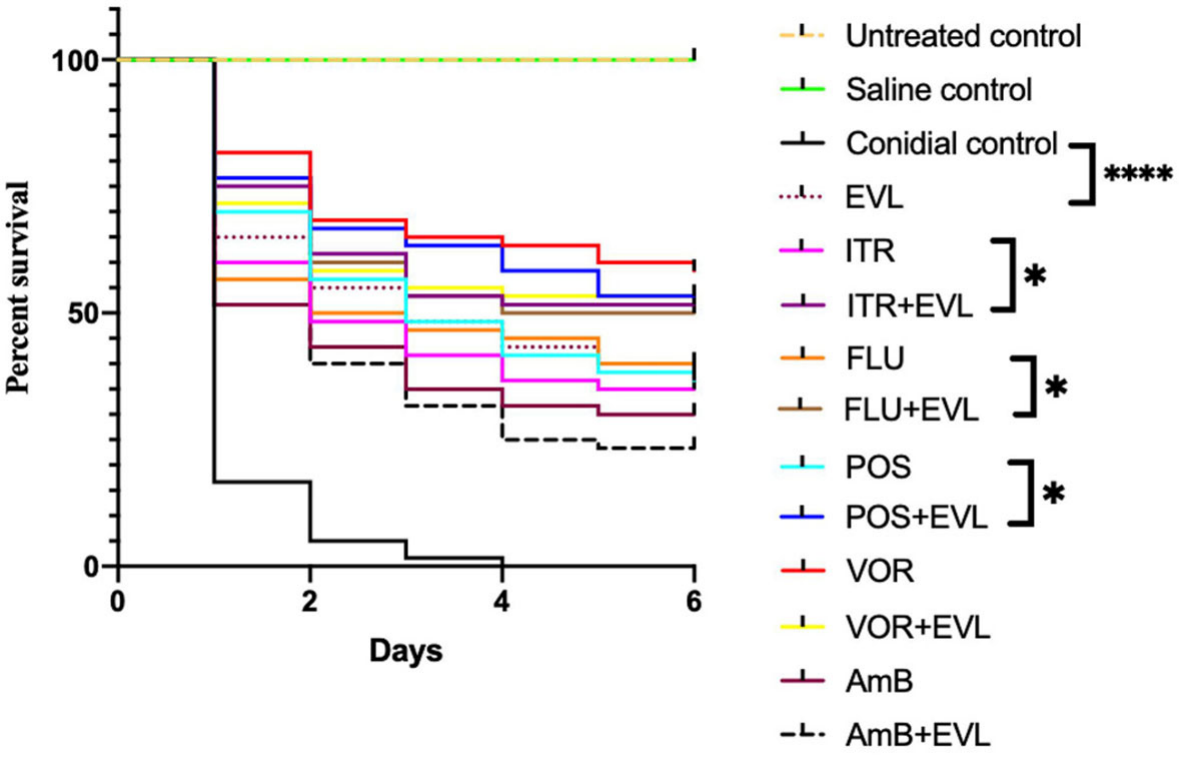
*Galleria mellonella* survival curves following infection with *C.neoformans*. Untreated group, noninfected larvae; Saline group, noninfected larvae injected with saline; Conidial group, *C. neoforman*-infected larvae without any treatment; POS, treatment of *C. neoformans* -infected larvae treated with posaconazole (POS) alone; POS+EVL, treatment of *C. neoformans* -infected larvae with POS and everolimus (EVL); ITR, treatment of *C. neoformans* -infected larvae with itraconazole alone; ITR+EVL, treatment of *C.neoformans*-infected larvae with ITR and EVL; VOR, treatment of *C. neoformans* - infected larvae with voriconazole alone; VOR+EVL, treatment of *C. neoformans* -infected larvae with VOR and EVL; FLU, treatment of *C. neoformans* -infected larvae with fluconazole alone; FLU+EVL, treatment of *C. neoformans* -infected larvae with FLU and EVL; AmB, treatment of *C. neoformans* -infected larvae with amphotericin B alone; AmB+EVL, treatment of *C. neoformans*-infected larvae with AmB and EVL; EVL, treatment of *C. neoformans* -infected larvae with EVL alone (*p<0.05. **** *p*<0.0001).

The larvae survival rates on day 6 in the POS, FLU, ITR, EVL, EVL+POS, EVL+FLU, and EVL+ITR groups were 36.7%, 38.3%, 35%, 40%, 53.3%, 50%, and 51.7%, respectively, confirming that EVL+POS, EVL+FLU, EVL+ITR treatment substantially increased larval survival in comparison to the POS group, FLU group, ITR group, and conidial group (P < 0.05) (**Figure 1**).

However, EVL-AmB and EVL-VOR combination did not show a synergistic effect when *G. mellonella* infected with *C.neoformans G7* isolate were treated, which was not consistent with *in vitro* experiments. It was speculated that the specific mechanism of drug interaction is different *in vivo* and *in vitro*.

## Discussion

Since its discovery, the TOR signaling pathway—of which TOR kinase is the primary component—has been the subject of many studies and has come to be regarded as a key regulator of cell proliferation in eukaryotes (Crespo and Hall, 2002). Rapamycin, a classical allosteric TOR inhibitor, was first found in screening for new antifungal drugs and has since shown great medicinal promise. Researchers have shown that rapamycin has strong antifungal effects against various fungi, including *Penicillium* spp., *Fusarium* spp., *Aspergillus* spp., *Cryptococcus* spp., *Candida* spp., *Dermatophytes* spp.(Rohde and Cardenas, 2004).However, rapamycin’s much more potent immunosuppressive properties prevented it from being employed as an antifungal agent (Gao et al., 2016).

Initially created to treat cancer, EVL was an orally active, potent TOR kinase inhibitor that directly inhibited tumor cell proliferation and growth and indirectly blocked angiogenesis (Hasskarl, 2018). We tested EVL’s antifungal potential against *Cryptococcus* spp. *in vitro*, alone and in combination with other antifungal drugs. The results discovered that EVL alone was active against all tested strains, which could be explained by the fact that FKBP protein (FK-506 Binding Proteins) and TOR protein are ubiquitous in eukaryotes, and rapalogs could bind to the FKBP12, and then, this complex inhibits mTOR to regulate the response of fungi to the external environment, and kills microorganism (Stan et al., 1994; Hasskarl, 2018). Furthermore, synergistic activities between EVL and AmB(88.9%), FLU(61.1%), ITR(55.6%), POS(50%), VOR(33.3%) were observed in Cryptococcus spp. The effective working ranges of EVL were 0.0625-0.5 μg/ml against *Cryptococcus* spp.; no antagonism was observed. The synergy between EVL and FLU was seen in as many as 61.1% of *Cryptococcus* strains, suggesting that EVL might increase the *in vitro* susceptibility of FLU-inactive *Cryptococcus* strains even when their MICs against FLU were high (0.5-32 μg/ml).

Rapamycin has been shown to have synergistic interactions with POS (40%), ITR (50%), and AmB (70%) against Mucorales (formerly called zygomycetes) by the broth microdilution checkerboard procedure (Dannaoui et al., 2009). Additionally, it has been reported that Mucorales exhibits antagonism of rapamycin/ITR, and no substantial agonism of rapamycin/POS (Dannaoui et al., 2009; Narreddy et al., 2010). In contrast, no antagonism was identified between EVL and POS or ITR in the current investigation, and EVL increased the antifungal activity of POS (50%) and ITR (55.6%). The different effect of EVL and rapamycin on TOR and antifungals, as well as the differential responsiveness of the tested species to these drugs, may account for these discrepancies.

Since *G. mellonella* larvae exhibit immunological responses comparable to those of mammals without the accompanying ethical concerns with advantages such as being easy to manipulate and inexpensive, these larvae have recently emerged as an appropriate *in vivo* model for the preclinical investigation of the antifungal effects of novel medicines (Vilcinskas, 2011; Favre-Godal et al., 2014; Liu et al., 2017). To further verify the interaction between EVL and azoles, EVL and AmB detected in our study, we tested the *in vivo* effect of this combination against one of the isolates of *C.neoformans* (G7) with the *G. mellonella* model. We found that EVL-POS, EVL-FLU, and ELV-ITR combination treatment were associated with significant improvements in larval survival compared to POS, FLU, or ITR treatment in isolation, further confirming the synergistic benefits of EVL-POS, ELV-FLU, and ELV-ITR combination treatment when used to treat infections caused by *C.neoformans* spp.

Ergosterol biosynthesis in fungal cell membranes was inhibited by azole antifungal compounds by inhibiting lanosterol 14α-demethylase activity (Keady and Thacker, 2005; Singal and Khanna, 2011; Jo Siu et al., 2013). Micropores in the cell membrane are formed, and the membrane’s permeability to monovalent and divalent cations is increased when ergosterol on the fungal cell membrane binds with AmB to form the sterol-polyene complex, killing fungus.

As an oral mTOR protein kinase inhibitor, EVL might control cell growth to kill fungi and potentiate the activities of azoles by inhibiting TOR signaling which could affect the amino acid permeases regulation, ribosome biogenesis, protein synthesis initiation, actin cytoskeleton organization, autophagy inhibition, control of phosphatases by TOR, and transcriptional control of nutrient metabolism(Crespo and Hall, 2002).

In conclusion, the current investigation expands prior results in the combination interactions between conventional antifungals and TOR inhibitors. Against *Cryptococcus* spp., EVL may augment the antifungal activity of AmB, POS, FLU, ITR, and VOR *in vitro*. In addition, for some individuals with clinical cancer, combining EVL with AmB or azoles may be a safe alternative for treating *Cryptococcus* infections. However, further research is required to clarify the underlying process and identify viable, safe therapeutic applications.

## Data availability statement

The original contributions presented in the study are included in the article/supplementary material. Further inquiries can be directed to the corresponding authors.

## Author contributions

PL conceived and designed the study. PL performed all the experiments, analyzed the data and wrote the manuscript. JS and QL provided general guidance and revised the manuscript. All authors contributed to the article and approved the submitted version.

## References

[B1] Amorim-Vaz S. Delarze E. Ischer F. Sanglard D. Coste A. T. (2015). Examining the virulence of candida albicans transcription factor mutants using galleria mellonella and mouse infection models. Front. Microbiol. 6. doi: 10.3389/fmicb.2015.00367 PMC4419840 25999923

[B2] Cho Y. J. Han S. I. Lim S. C. (2021). Cryptococcal infection presenting as soft tissue abscess and arthritis: Case report. Med. (Baltimore). 100 (28), e26656. doi: 10.1097/MD.0000000000026656 PMC8284701 34260570

[B3] Clinical and Laboratory Standards Institute (2017). Reference Method for Broth Dilution Antifungal Susceptibility Testing of Yeasts; Approved Standard-4th ed CLSI Document M27-A4.

[B4] Crespo J. L. Hall M. N. (2002). Elucidating TOR signaling and rapamycin action: lessons from saccharomyces cerevisiae. Microbiol. Mol. Biol. Rev. 66 (4), 579–591. doi: 10.1128/MMBR.66.4.579-591.2002 12456783 PMC134654

[B5] Cruz M. C. Cavallo L. M. Görlach J. M. Cox G. Perfect J. R. Cardenas M. E.. (1999). Rapamycin antifungal action is mediated *via* conserved complexes with FKBP12 and TOR kinase homologs in cryptococcus neoformans. Mol. Cell Biol. 19 (6), 4101–4112. doi: 10.1128/MCB.19.6.4101 10330150 PMC104369

[B6] Dannaoui E. Schwarz P. Lortholary O. (2009). *In vitro* interactions between antifungals and immunosuppressive drugs against zygomycetes. Antimicrob. Agents Chemother. 53 (8), 3549–3551. doi: 10.1128/AAC.00184-09 19451295 PMC2715618

[B7] DiNardo A. R. Schmidt D. Mitchell A. Kaufman Y. Tweardy D. J. (2015). First description of oral cryptococcus neoformans causing osteomyelitis of the mandible, manubrium and third rib with associated soft tissue abscesses in an immunocompetent host. Clin. Microbiol. Case Rep. 1 (3), 017. 27227123 PMC4876986

[B8] Favre-Godal Q. Dorsaz S. Queiroz E. F. Conan C. Marcuort L. Eko Wardojo B. P.. (2014). Comprehensive approach for the detection of antifungal compounds using a susceptible strain of candida albicans and confirmation of *in vivo* activity with the galleria mellonella model. Phytochemistry 105, 68–78. doi: 10.1016/j.phytochem.2014.06.004 24984572

[B9] Fonseca F. L. Reis F. C. G. Sena B. A. G. Jozefowicz L. J. Kmetzsch L. Rodrigues M. L. (2019). The overlooked glycan components of the cryptococcus capsule. Curr. Top. Microbiol. Immunol. 422, 31–43. doi: 10.1007/82_2018_140 30203395

[B10] Gao L. Sun Y. He C. Li M. Zeng T. Lu Q. (2016). INK128 exhibits synergy with azoles against exophiala spp. and fusarium spp. Front. Microbiol. 7. doi: 10.3389/fmicb.2016.01658 PMC5071350 27812353

[B11] Hasskarl J. (2018). Everolimus. Recent Results Cancer Res. 211, 101–123. doi: 10.1007/978-3-319-91442-8_8 30069763

[B12] Henao-Martínez A. F. Beckham J. D. (2015). Cryptococcosis in solid organ transplant recipients. Curr. Opin. Infect. Dis. 28 (4), 300–307. doi: 10.1097/QCO.0000000000000171 26098495

[B13] High K. P. Washburn R. G. (1997). Invasive aspergillosis in mice immunosuppressed with cyclosporin a, tacrolimus (FK506), or sirolimus (rapamycin). J. Infect. Dis. 175 (1), 222–225. doi: 10.1093/infdis/175.1.222 8985226

[B14] Jo Siu W. J. Tatsumi Y. Senda H. Pillai R. Nakamura T. Sone D.. (2013). Comparison of *in vitro* antifungal activities of efinaconazole and currently available antifungal agents against a variety of pathogenic fungi associated with onychomycosis. Antimicrob. Agents Chemother. 57 (4), 1610–1616. doi: 10.1128/AAC.02056-12 23318803 PMC3623347

[B15] Keady S. Thacker M. (2005). Voriconazole in the treatment of invasive fungal infections. Intensive Crit. Care Nurs. 21 (6), 370–373. doi: 10.1016/j.iccn.2005.02.006 15985371

[B16] Liu X. Li T. Wang D. Yang Y. Sun W. Liu J.. (2017). Synergistic antifungal effect of fluconazole combined with licofelone against resistant candida albicans. Front. Microbiol. 8. doi: 10.3389/fmicb.2017.02101 PMC5681995 29163396

[B17] Maziarz E. K. Perfect J. R. (2016). Cryptococcosis. Infect. Dis. Clin. North Am. 30 (1), 179–206. doi: 10.1016/j.idc.2015.10.006 26897067 PMC5808417

[B18] Mylonakis E. Moreno R. El Khoury J. B. Idnurm A. Heitman J. Calderwood S. B.. (2005). Galleria mellonella as a model system to study cryptococcus neoformans pathogenesis. Infect. Immun. 73 (7), 3842–3850. doi: 10.1128/IAI.73.7.3842-3850.2005 15972469 PMC1168598

[B19] Narreddy S. Manavathu E. Chandrasekar P. H. Alangaden G. J. Revankar S. G. (2010). *In vitro* interaction of posaconazole with calcineurin inhibitors and sirolimus against zygomycetes. J. Antimicrob. Chemother. 65 (4), 701–703. doi: 10.1093/jac/dkq020 20130026

[B20] Odds F. C. (2003). Synergy, antagonism, and what the chequerboard puts between them. J. Antimicrob. Chemother. 52 (1), 1. doi: 10.1093/jac/dkg301 12805255

[B21] Pappas P. G. Perfect J. R. Cloud G. A. Larsen R. A. Pankey G. A. Lancaster D. J.. (2001). Cryptococcosis in human immunodeficiency virus-negative patients in the era of effective azole therapy. Clin. Infect. Dis. 33 (5), 690–699. doi: 10.1086/322597 11477526

[B22] Perfect J. R. Dismukes W. E. Dromer F. Goldman D. L. Graybill J. R. Hamill R. J.. (2010). Clinical practice guidelines for the management of cryptococcal disease: 2010 update by the infectious diseases society of america. Clin. Infect. Dis. 50 (3), 291–322. doi: 10.1086/649858 20047480 PMC5826644

[B23] Rohde J. R. Cardenas M. E. (2004). Nutrient signaling through TOR kinases controls gene expression and cellular differentiation in fungi. Curr. Top. Microbiol. Immunol. 279, 53–72. doi: 10.1007/978-3-642-18930-2_4 14560951

[B24] Schwarz P. Dannaoui E. (2020). *In vitro* interaction between isavuconazole and tacrolimus, cyclosporin a, or sirolimus against aspergillus species. J. Fungi (Basel). 6 (3), 103. doi: 10.3390/jof6030103 32650564 PMC7560155

[B25] Singal A. Khanna D. (2011). Onychomycosis: Diagnosis and management. Indian J. Dermatol. Venereol. Leprol. 77 (6), 659–672. doi: 10.4103/0378-6323.86475 22016272

[B26] Singh N. Forrest G. (2009). AST infectious diseases community of practice. cryptococcosis in solid organ transplant recipients. Am. J. Transplant. 9 Suppl 4, S192–S198. doi: 10.1111/j.1600-6143.2009.02911.x 20070681

[B27] Stan R. McLaughlin M. M. Cafferkey R. Johnson R. K. Rosenberg M. Livi G. P. (1994). Interaction between FKBP12-rapamycin and TOR involves a conserved serine residue. J. Biol. Chem. 269 (51), 32027–32030. 7528205

[B28] Teichert S. Wottawa M. Schönig B. Tudzynski B. (2006). Role of the fusarium fujikuroi TOR kinase in nitrogen regulation and secondary metabolism. Eukaryot. Cell. 5 (10), 1807–1819. doi: 10.1128/EC.00039-06 17031002 PMC1595341

[B29] Thakur M. Revankar S. G. (2011). *In vitro* interaction of caspofungin and immunosuppressives against agents of mucormycosis. J. Antimicrob. Chemother. 66 (10), 2312–2314. doi: 10.1093/jac/dkr297 21795260

[B30] Vézina C. Kudelski A. Sehgal S. N. (1975). Rapamycin (AY-22,989), a new antifungal antibiotic. i. taxonomy of the producing streptomycete and isolation of the active principle. J. Antibiot. (Tokyo). 28 (10), 721–726. doi: 10.7164/antibiotics.28.721 1102508

[B31] Vilcinskas A. (2011). Insects emerge as valuable model hosts to explore virulence. Virulence 2 (5), 376–378. doi: 10.4161/viru.2.5.18289 22015622

